# Supplementary material for: The molecular basis for acetylhistidine synthesis by HisAT/NAT16
Source: Nat Commun. 2025 Jul 1;16:5960. doi: 10.1038/s41467-025-61145-x (PMC12219266; doi:10.1038/s41467-025-61145-x)
Supplement: Supplementary file 1 — Supplementary Information [file 41467_2025_61145_MOESM1_ESM.pdf]

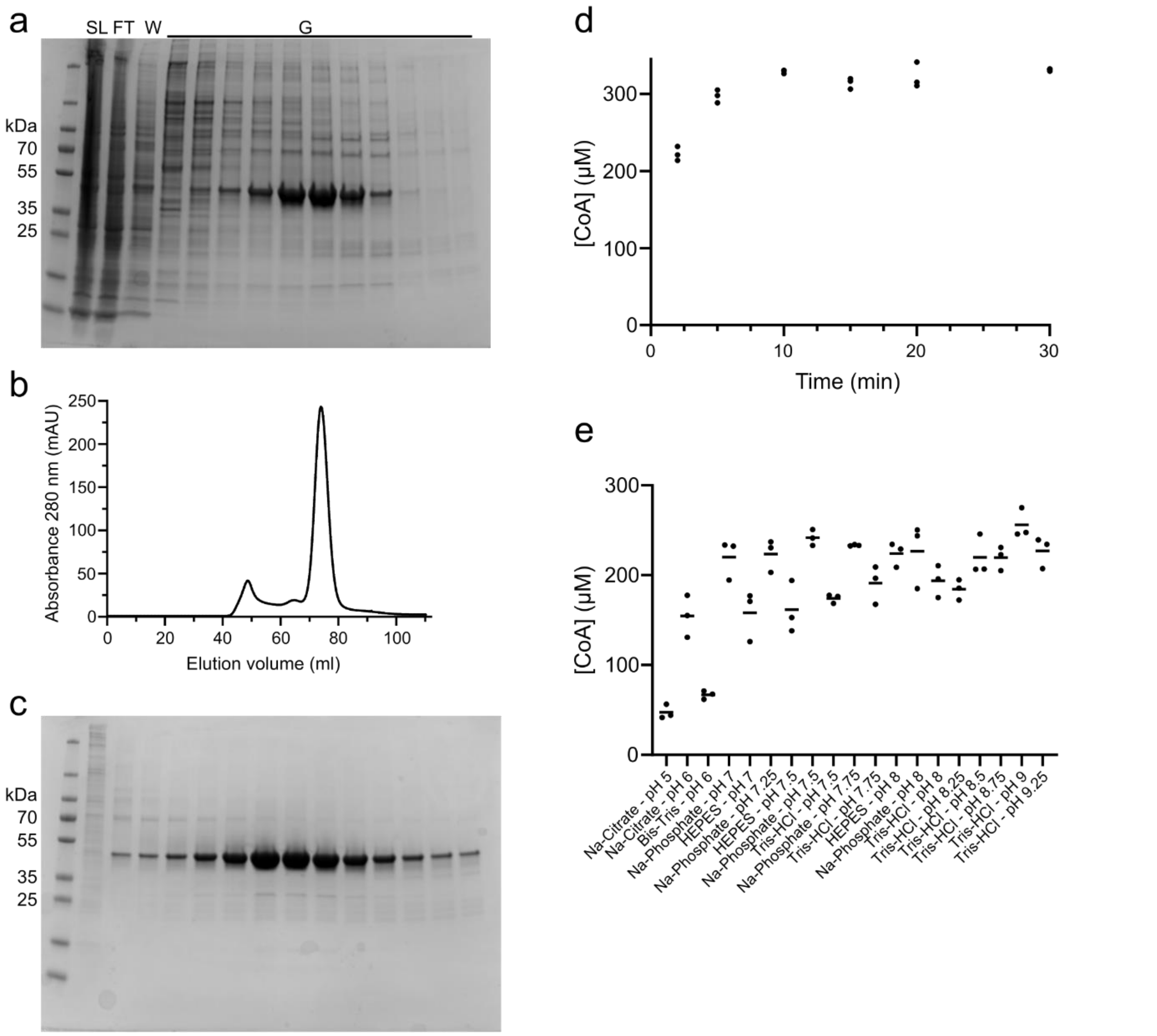

Supplementary Figure 1. Protein purification, DTNB assay time curve and pH/buffer screen. a) Coomassie-stained SDS-PAGE of the fractions from a Ni^{2+}-affinity purification of HisAT from insect cell lysate. SL = soluble lysate, FT = flow-through, W = wash, G = fractions from the imidazole elution gradient. b) Size-exclusion chromatogram of an elution from Superdex 75 16/60 column, and c) SDS-PAGE of the fractions from this elution. d) Time course of a HisAT DTNB assay with Ac-CoA and histidine with three samples and one control (without enzyme) per time point. The individual data points after control subtraction are shown. e) Buffer and pH screening of HisAT activity using a DTNB assay with histidine and Ac-CoA, 10-minute reaction time, with three technical replicates and one control (without enzyme) per condition. The data points for the three samples and their mean. Source data are provided as a Source Data file.

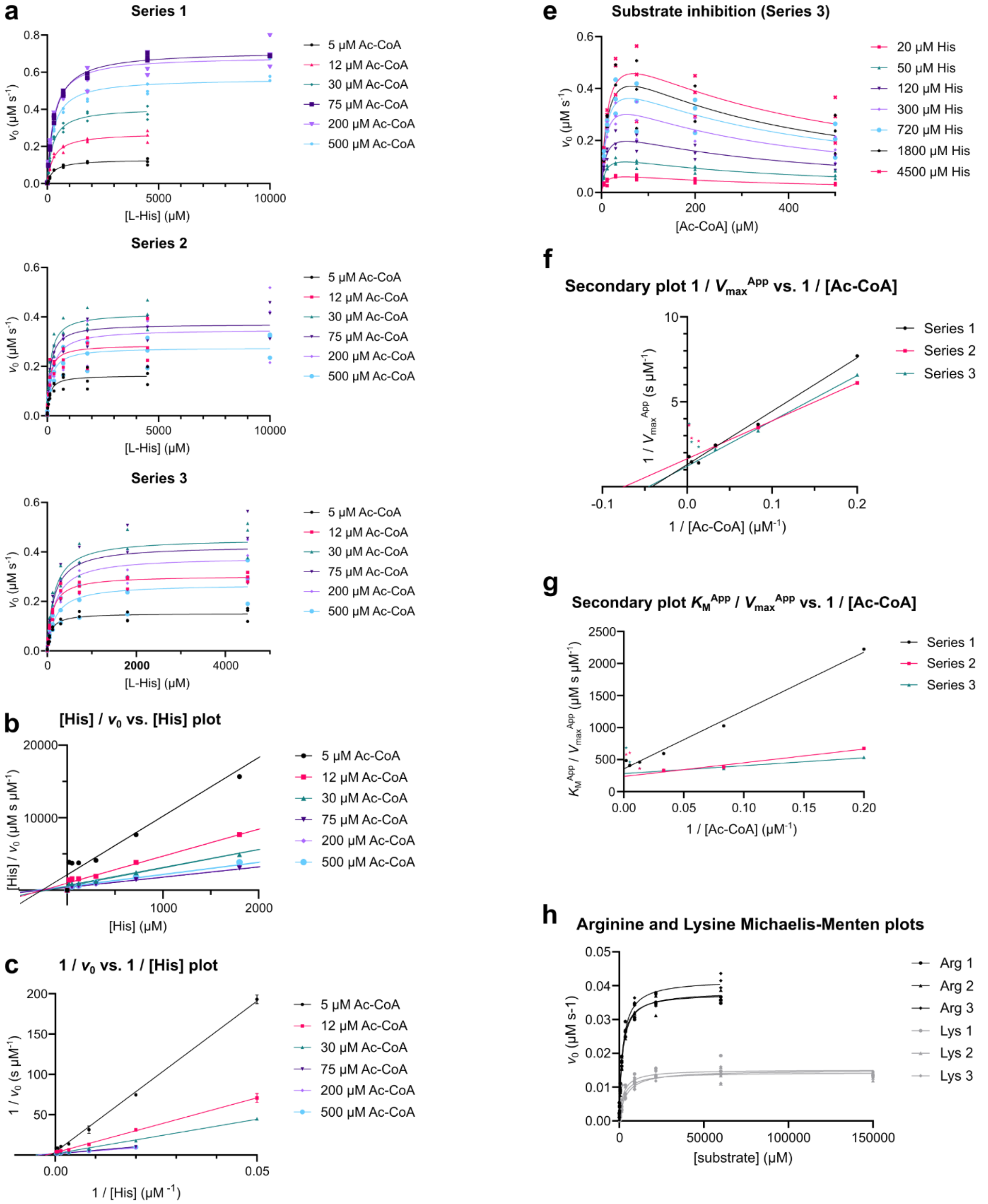

d

		[Ac-CoA] (μM) 5	12	30	75	200	500
Series 1	V_{max}^{App} (μM s^{-1})	0.1299	0.2724	0.4085	0.7135	0.6852	0.5655
	K_M^{App} (μM)	288.8	279.4	242.8	328.2	280.8	273.9
Series 2	V_{max}^{App} (μM s^{-1})	0.1635	0.2864	0.4149	*0.3711*	*0.3497*	*0.2759*
	K_M^{App} (μM)	110.1	109.3	136.9	*134.1*	*211.7*	*160.0*
Series 3	V_{max}^{App} (μM s^{-1})	0.1518	0.3036	0.4538	*0.4254*	*0.3794*	*0.2693*
	K_M^{App} (μM)	81.01	109.9	153.1	*154.7*	*175.0*	*184.4*

i

	Arginine		Lysine	
	Mean	St.Dev.	Mean	St.Dev.
k_{cat}^{App} (s^{-1})	0.785	0.036	0.297	0.007
K_M^{App} (μm)	1853	168	3007	764
k_{cat}/K_M (s^{-1} μM^{-1})	0.000426	0.000025	0.000105	0.000025

Supplementary Figure 2. Initial velocities, primary and secondary plots from the enzyme kinetics assays. a) Plots of the initial velocities and the Michelis-Menten equation

fits for the three separate assay series of variable histidine concentrations (0, 20, 50, 120, 300, 720, 1800, or 4500 µM for the three lower Ac-CoA concentrations in Series 1 and 2 and for all Ac-CoA concentrations in Series 3, and 0, 50, 120, 300, 720, 1800, 4500, or 10000 µM for the three higher Ac-CoA concentrations in Series 1 and 2) at different fixed Ac-CoA concentrations indicated in the legend. HisAT concentration was 20 nM. b) Plot of the initial rates from Series 1 on [His] / v_0 vs. [His] plot showing the lines at different Ac-CoA concentration meeting to the left of the Y-axis suggesting a ternary complex mechanism. The points shown are the mean of the three parallels. The axes have been truncated for clarity. c) Plot of the initial rates from Series 1 on 1 / v_0 vs. 1 / [His] plot showing the lines representing different Ac-CoA concentration are non-parallel and intercept each other at the Y-axis suggesting a ternary complex mechanism. The points shown are the mean of the three parallels. d) The apparent V_{max} and K_M values that were determined with variable histidine concentrations at different fixed Ac-CoA concentrations. The values were determined from the fits to the initial velocity plots in panel a. e) The data from series 3 reversed to show the effect of Ac-CoA concentration on the initial rate at different histidine concentrations and plotted including the substrate inhibition using the formula $v_0 = V_{max}$ [Ac-CoA] / (K_M + [Ac-CoA] (1+[Ac-CoA] / K_i)). The mean K_i for Ac-CoA determined based on these fits was 333 µM (St.Dev. 19 µM). f) The double-reciprocal secondary plots of 1 / V_{max}^{App} vs. 1 / [Ac-CoA] generated using the apparent V_{max} values derived from the fits of Supplementary Figure 2a and listed in Supplementary Figure 2c. V_{max} was derived from the inverse of the Y-axis intercept and $K_M^{Ac\text{-}CoA}$ from the negative inverse of the X-axis intercept of these plots. g) The double-reciprocal secondary plot of K_M^{App} / V_{max}^{App} vs. 1 / [Ac-CoA]. This plot was used to determine, from the Y-intercept, K_M^{His} / V_{max}, from which K_M^{His} was calculated by multiplication with V_{max} derived from Supplementary Figure 2d. The values in cursive in panel c and marked with star in panels d and e were not used in fitting of the lines used to estimate the kinetic constants due to the substrate inhibition elevating the values. h) The plots of the initial velocities and the Michaelis-Menten equation fit for the three separate assays with variable arginine (0, 240, 600, 1440, 3600, 9000, 21600, or 60000 µM) and lysine (0, 600, 1440, 3600, 9000, 21600, 60000, or 150000 µM) with fixed Ac-CoA (50 µM). HisAT concentration here was 50 nM. i) The apparent k_{cat}, K_M, and k_{cat} / K_M values for arginine and lysine determined from the three independent experiments plotted in panel h. Source data are provided as a Source Data file.

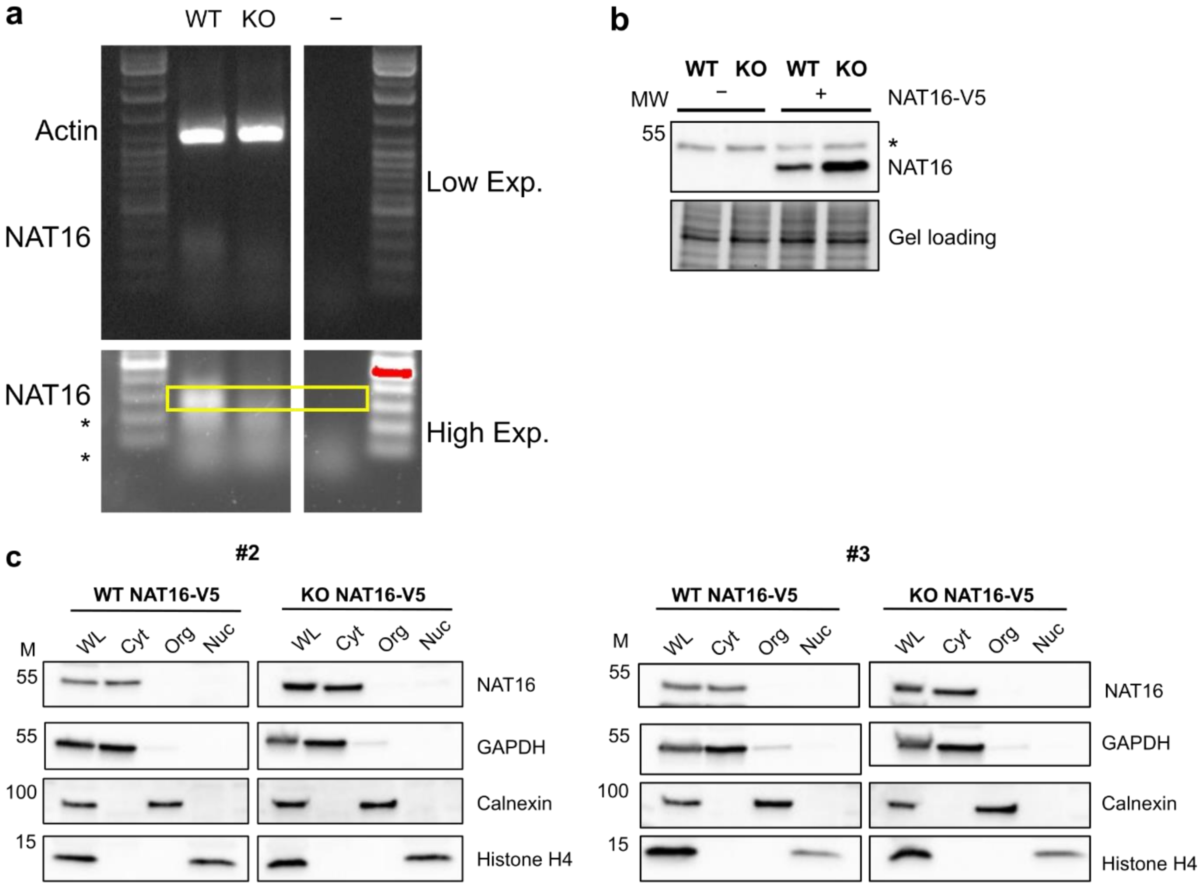

Supplementary Figure 3. Verification of NAT16 KO and the subcellular localization assay. a) Verification of NAT16 KO cells. RT-PCR of RNA isolated from HAP1 WT and NAT16 KO cells using NAT16 primer set overlapping with the 4nt deletion in the NAT16 ORF. The NAT16 band is highlighted with a yellow box. Actin was used as a loading control. Negative control without RNA was marked with minus sign. Additional lanes on the agarose gel between the 'KO' and '–' lanes have been removed. b) Western blot analysis of HAP1 WT cells (WT) and HAP1 NAT16 KO cells (KO) with and without NAT16-V5 overexpression. Anti-NAT16 detected only overexpressed NAT16. * marks an unspecific band. Gel loading was included as a control. c) Two additional independent experiments of HAP1 cell fractionation with WT and NAT16 KO cells both with NAT16-V5 overexpression. The first experiment is shown in Figure 2b. Western blotting analysis of whole lysate (WL), cytosolic fraction (Cyt), organellar fraction (Org) and the nuclear fraction (Nuc). Different antibodies were included as controls for the different subcellular compartments. Anti-GAPDH as a cytosolic control, anti-Calnexin an organellar control and anti-Histone H4 a nuclear control. Anti-V5 was used to detect NAT16-V5. Source data are provided as a Source Data file.

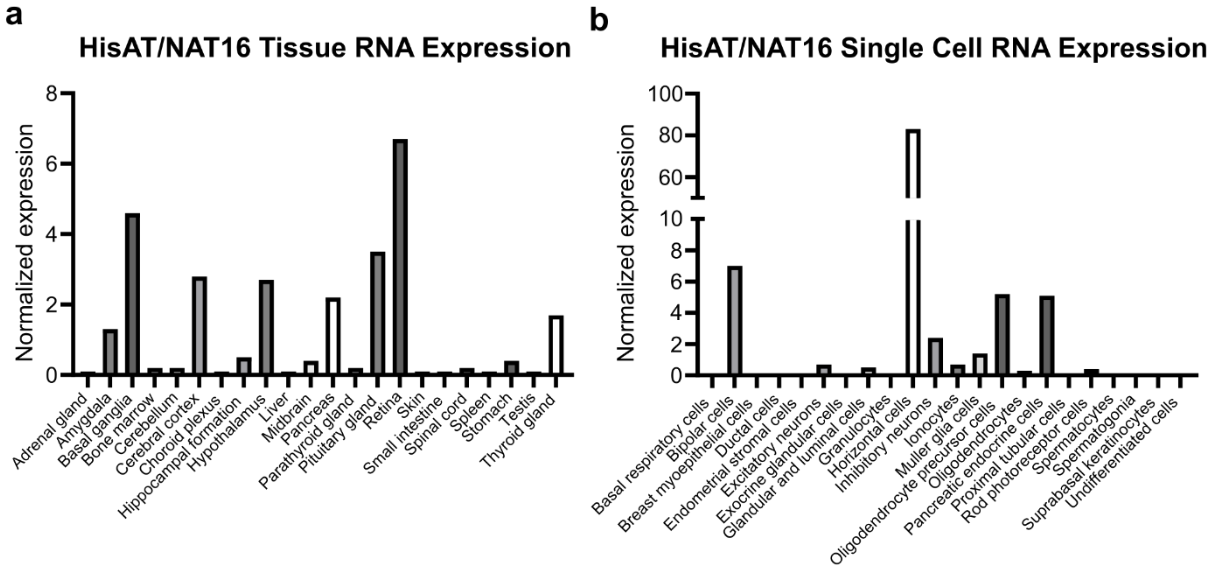

Supplementary Figure 4. HisAT/NAT16 expression level. HisAT/NAT16 mRNA expression levels a) in different human tissues and b) in different cells. The transcript data are based on Human Protein Atlas and Genotype-Tissue Expression project transcriptomics data available at the Human Protein Atlas website (v23.proteinatlas.org/ENSG00000167011-NAT16). Only tissues and cells with nonzero expression are shown. Source data are provided as a Source Data file.

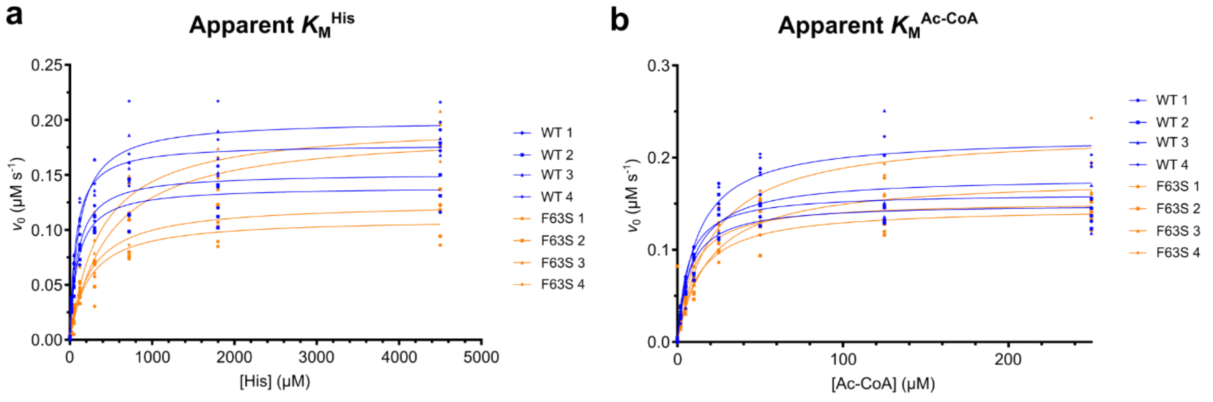

c

		WT		F63S	
		Mean	St.Dev.	Mean	St.Dev.
Variable Ac-CoA	k_{cat} (s^{-1})	8.91	1.38	8.80	1.61
	K_M (μM)	8.61	2.10	15.4	4.8
Variable His	k_{cat} (s^{-1})	8.35	1.17	7.78	1.93
	K_M (μM)	92.1	13.8	332	98

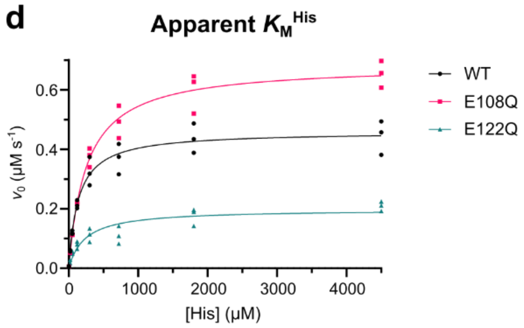

e

	WT	E108Q	E122Q
k_{cat} (s^{-1})	23.0	34.2	9.89
K_M (His) (μM)	138	257	234

Supplementary Figure 5. Activity comparison between HisAT WT and F63S and between WT and E108Q and E122Q. Plots of the initial velocities from the four independent assays with WT and F63S enzymes and the Michaelis-Menten equation fits for them with a) variable histidine (0, 20, 50, 120, 300, 720, 1800, or 4500 μM histidine with fixed 50 μM Ac-CoA) and b) variable Ac-CoA (0, 2, 5, 10, 25, 50, 125, or 250 μM Ac-CoA with fixed 5 mM histidine). c) The mean and standard deviation of the apparent kinetics constants for the WT and F63S enzymes. d) Plots of the initial velocities from a single assay with WT, E108Q, and E122Q enzymes (0, 20, 50, 120, 300, 720, 1800, or 4500 μM histidine with fixed 50 μM Ac-CoA), and e) the apparent kinetic constants from these assays. Source data are provided as a Source Data file.

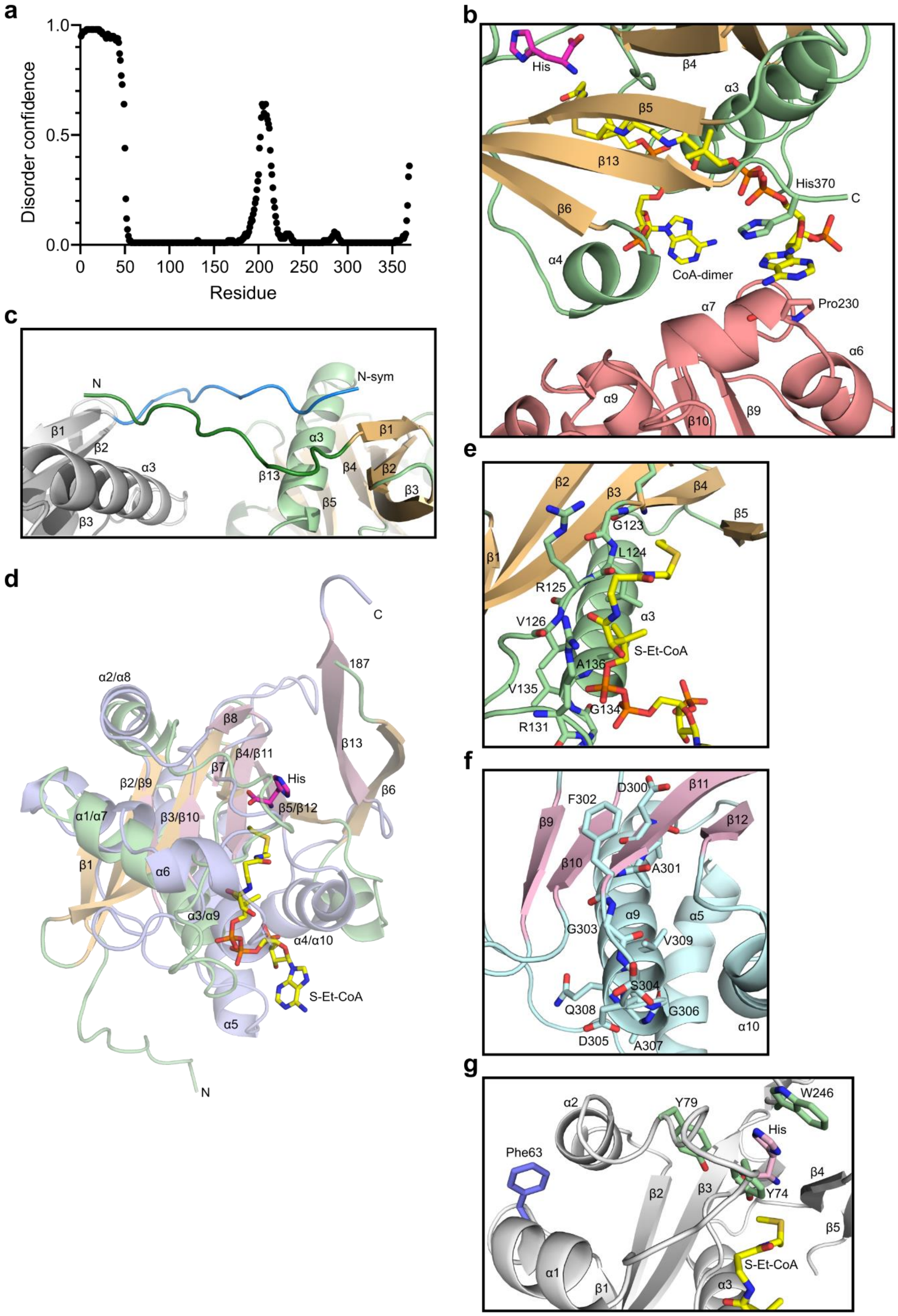

Supplementary Figure 6. Disorder prediction, crystal contacts, the double-GNAT fold, and Phe63. a) Plot of the predicted disordered regions in the HisAT sequence obtained with

DISOPRED3. Source data are provided as a Source Data file. b) A figure showing the crystal contact mediated by the CoA-disulfide between the first histidine (370) of the 6xHis-tag in one molecule and Pro230 in another molecule in the $P6_3$ crystals. The CoA-disulfide is shown in yellow, while the two HisAT molecules are shown in green/brown and red. c) Interaction of the truncated N-termini in the *I*222 crystals. CoA molecules are shown in yellow, one HisAT molecule is shown as green/brown with green N-terminus and the other as white with blue N-terminus. d) Overlay of the two GNAT domains with the first domain shown in green/brown and the second domain in blue/magenta. Histidine is shown in magenta and S-ethyl-CoA in yellow. e) The Ac-CoA binding region of the first GNAT domain and f) the corresponding region in the second GNAT domain. g) Showing the location of Phe63 in the α1 region of HisAT in the vicinity of the active site.

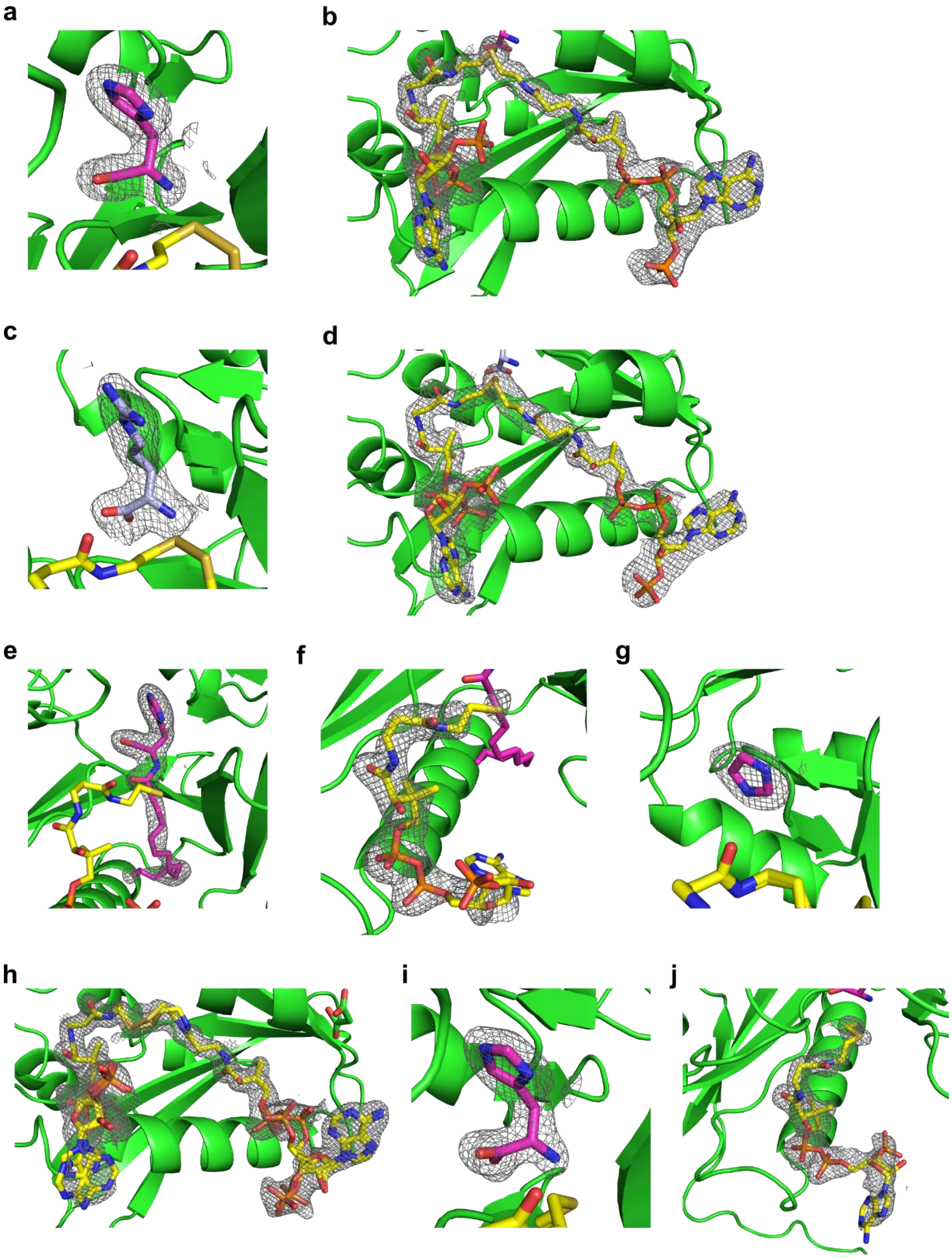

Supplementary Figure 7. Ligand 2Fo-Fc maps contoured at 1σ for a) and b) the structure with histidine (magenta) and CoA-disulfide (yellow), c) and d) for the structure with arginine (light blue) and CoA-disulfide (yellow), e) and f) for the structure with myristoyl histidine (magenta) and CoA (yellow), g) and h) for the structure with imidazole (magenta) and CoA-disulfide (yellow), and i) and j) for the structure with histidine (magenta) and S-ethyl-CoA (yellow).

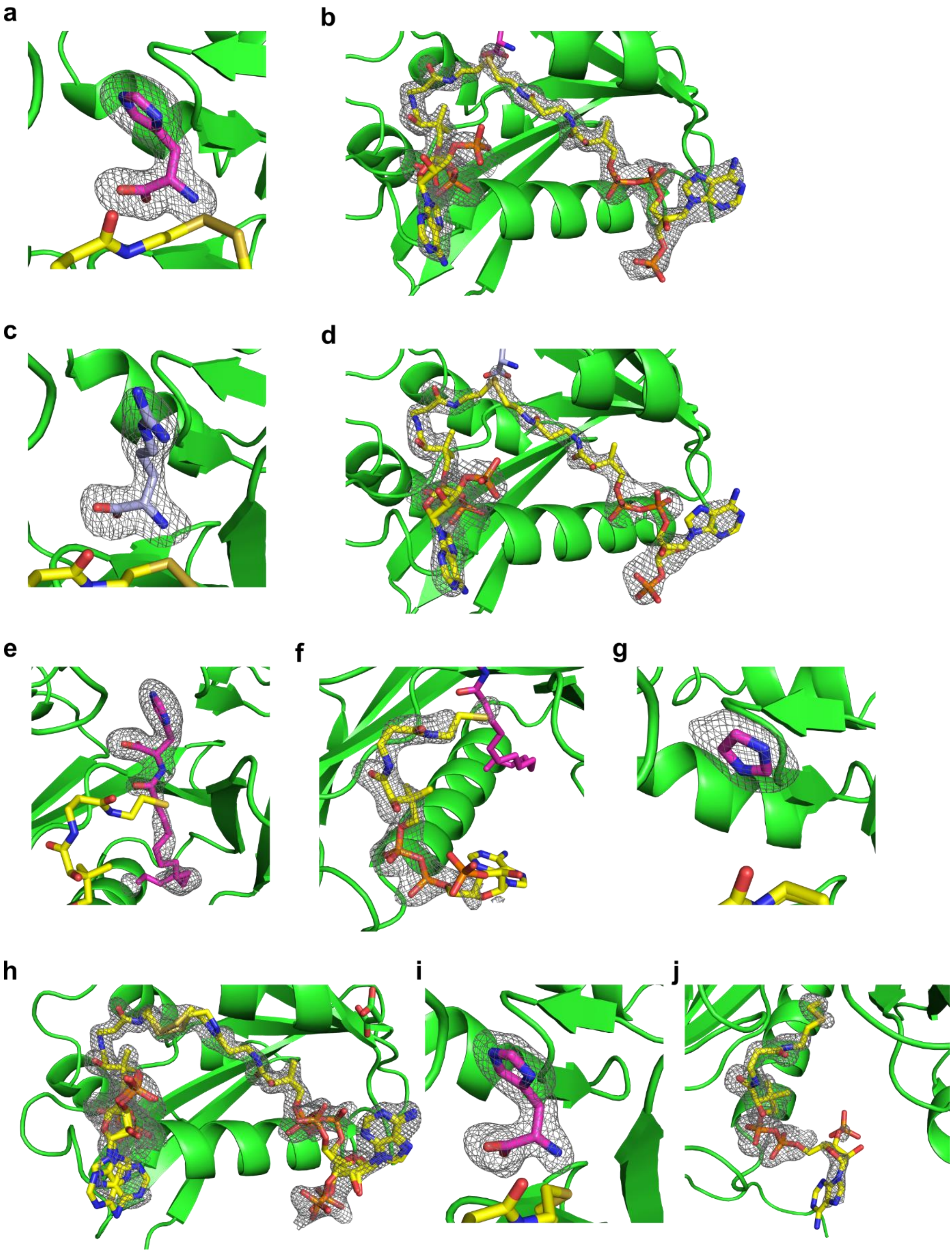

Supplementary Figure 8. Ligand omit Fo-Fc maps contoured at 3σ generated with phenix.polder for a) and b) the structure with histidine (magenta) and CoA-disulfide (yellow), c) and d) for the structure with arginine (light blue) and CoA-disulfide (yellow), e) and f) for the structure with myristoyl histidine (magenta) and CoA (yellow), g) and h) for the

structure with imidazole (magenta) and CoA-disulfide (yellow), and i) and j) for the structure with histidine (magenta) and S-ethyl-CoA (yellow).

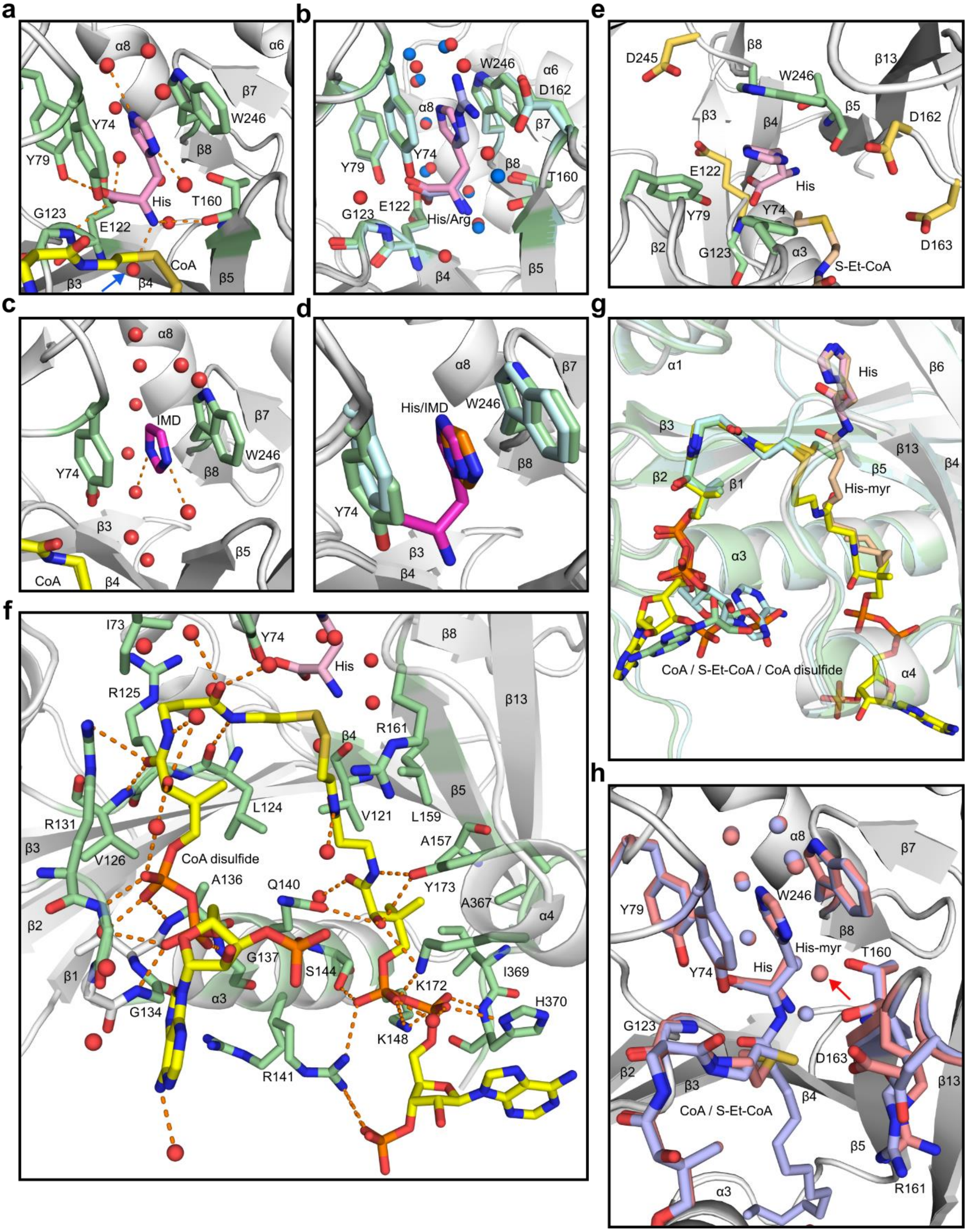

Supplementary Figure 9. Additional figures of the ligands bound to HisAT. a) Histidine-associated water structure in the presence of CoA-disulfide. Histidine is shown as pink and CoA-disulfide as yellow. Waters are presented as red spheres. The additional water that is not present in the structure with S-ethyl-CoA is highlighted with a blue arrow. b) Comparison of histidine (pink) and arginine (blue) binding position and respective water structures. Waters from histidine structure are red spheres and those from arginine structure blue spheres. c) Imidazole (magenta) binding in the HisAT active site. Waters are shown as red spheres. d)

Comparison of histidine (magenta) and imidazole (orange) binding in the HisAT active site. e) Acidic residues (yellow) near the substrate binding site. Histidine is shown in pink and S-ethyl-CoA in light brown. f) Binding mode of the CoA disulfide (yellow). Histidine is shown in pink, the interacting HisAT residues in green and waters as red spheres. g) Comparison of S-ethyl-CoA (light green), CoA (light blue), and CoA disulfide (yellow) binding and histidine (pink) and histidine myristate (light brown) binding. h) Comparison of substrate histidine and product myristoylhistidine binding. Histidine, S-ethyl-CoA, active site residues, and waters from the substrate structure are shown in pink and the myristoylhistidine, CoA, active site residues, and waters from the product structure are shown in blue. The water molecule missing from the product structure is highlighted with a red arrow.

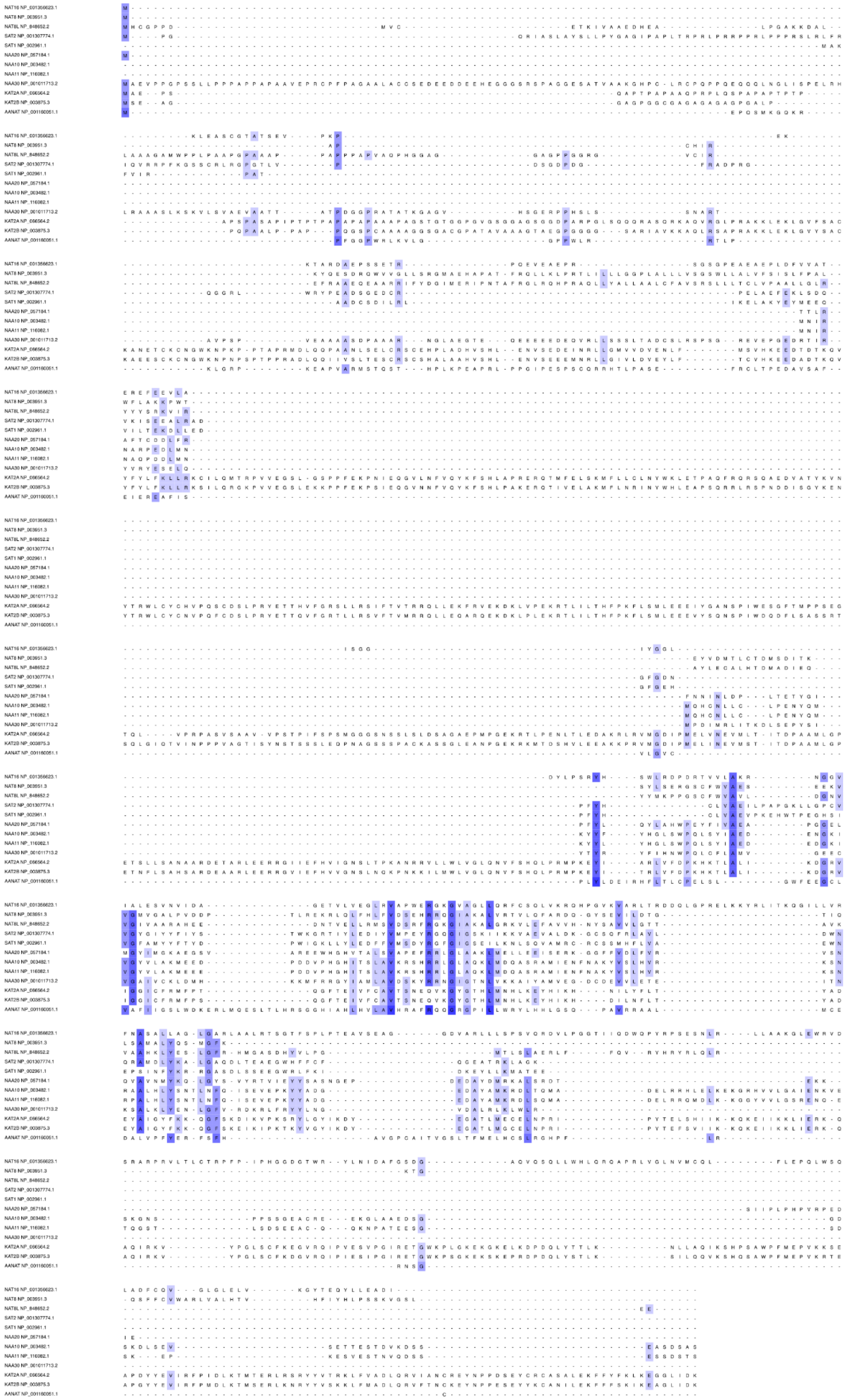
NAT16 NP_001356623.1
NAT8 NP_003951.3
NAT8L NP_848652.2
SAT2 NP_001307774.1
SAT1 NP_002961.1
NAA20 NP_057184.1
NAA10 NP_003482.1
NAA11 NP_116082.1
NAA30 NP_001011713.2
KAT2A NP_066564.2
KAT2B NP_003875.3
AANAT NP_001160051.1

Supplementary Figure 10. Multiple sequence alignment of 12 human GNAT proteins. Source data (sequences) are provided as a Source Data file. Darker color in a column indicates a more conserved position.

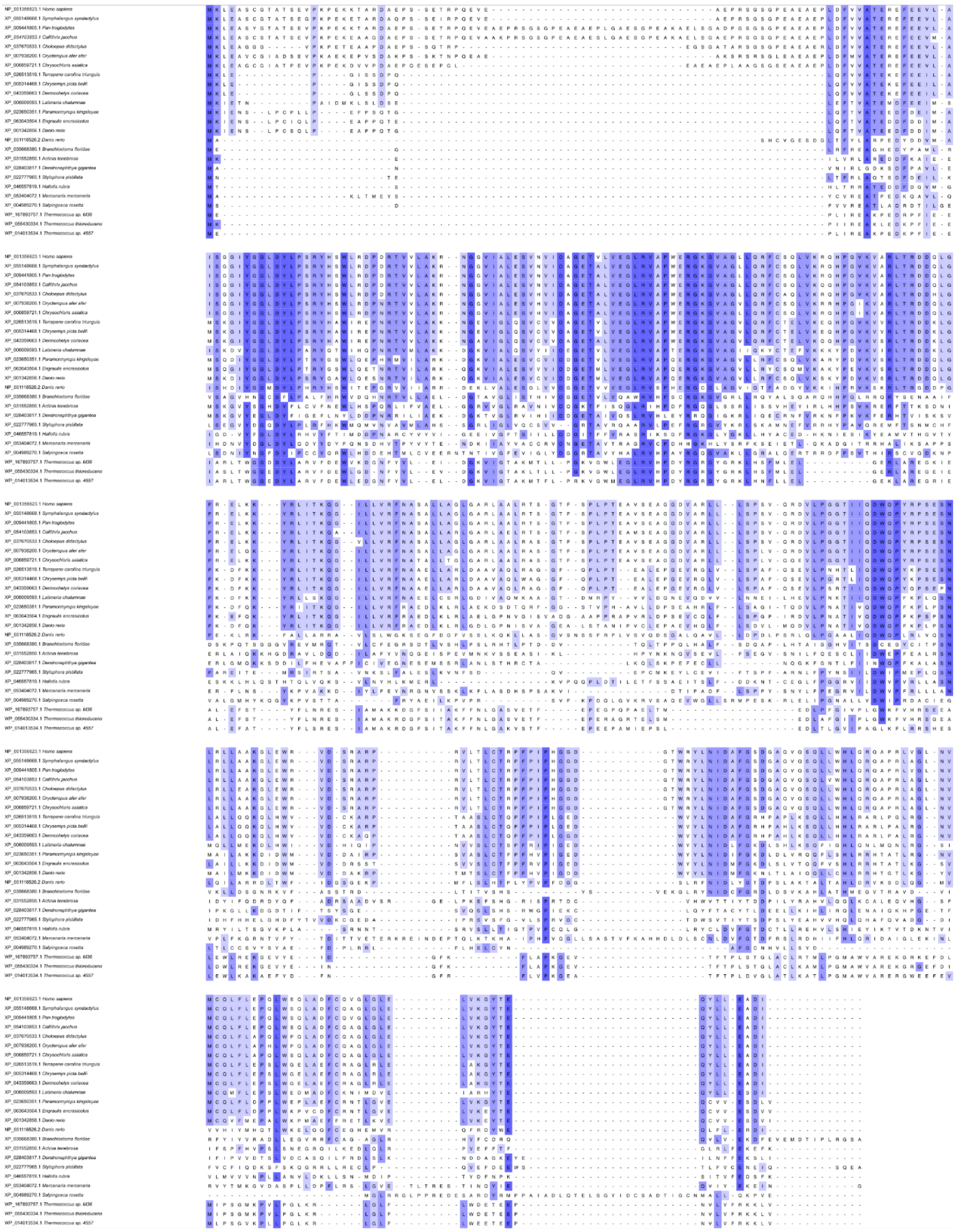

Supplementary Figure 11. Multiple sequence alignment of selected NAT16-like sequences from animals, a non-animal eukaryote, and archaea. Source data (sequences) are provided as a Source Data file. Darker color in a column indicates a more conserved position.

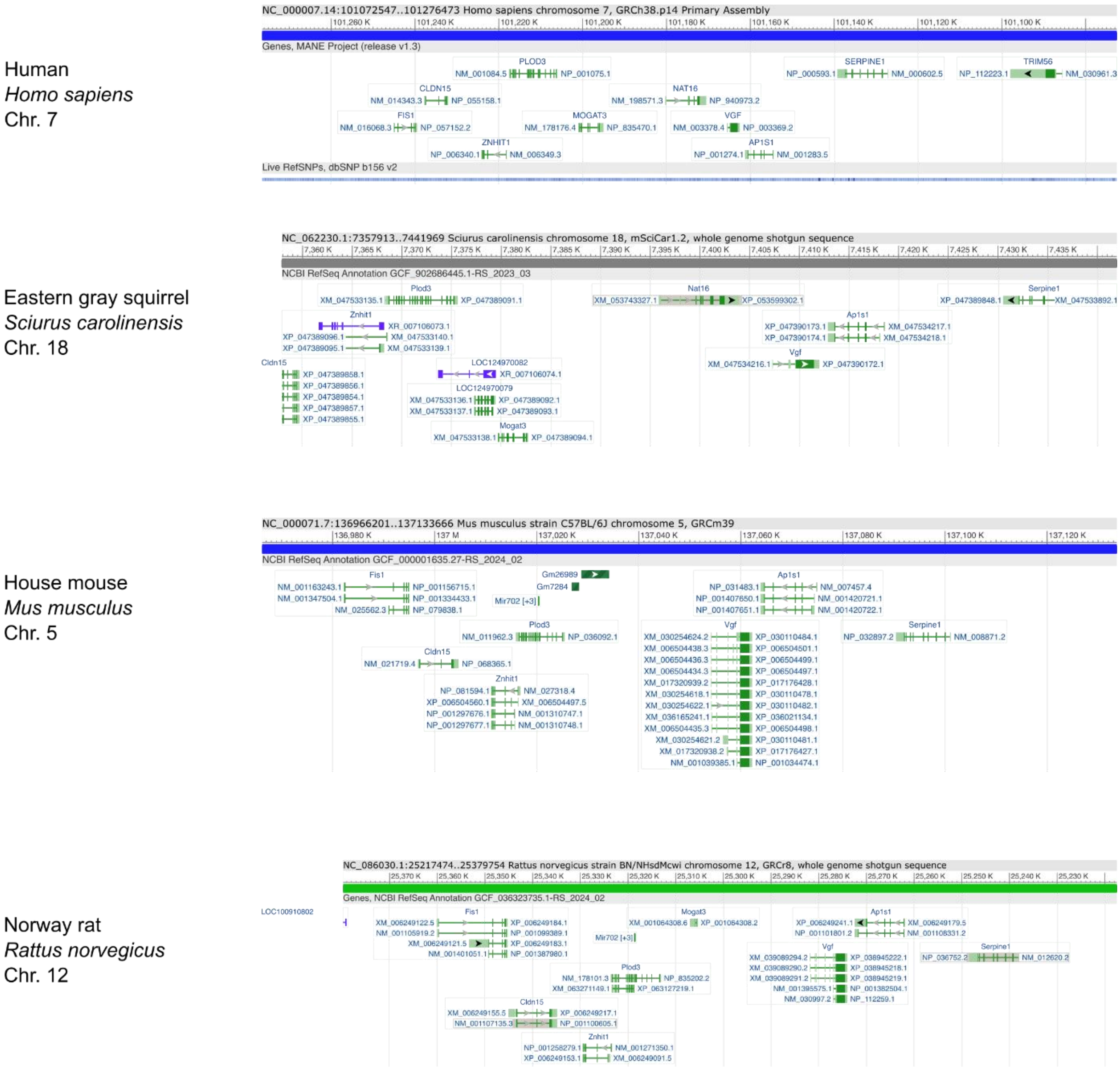

Supplementary Figure 12. Genome organization of human and squirrel around the *NAT16* gene, and the corresponding locus in mouse and rat that are missing the gene. Genome organizations were downloaded from the NCBI Gene database.

a) WT + NAT16-V5

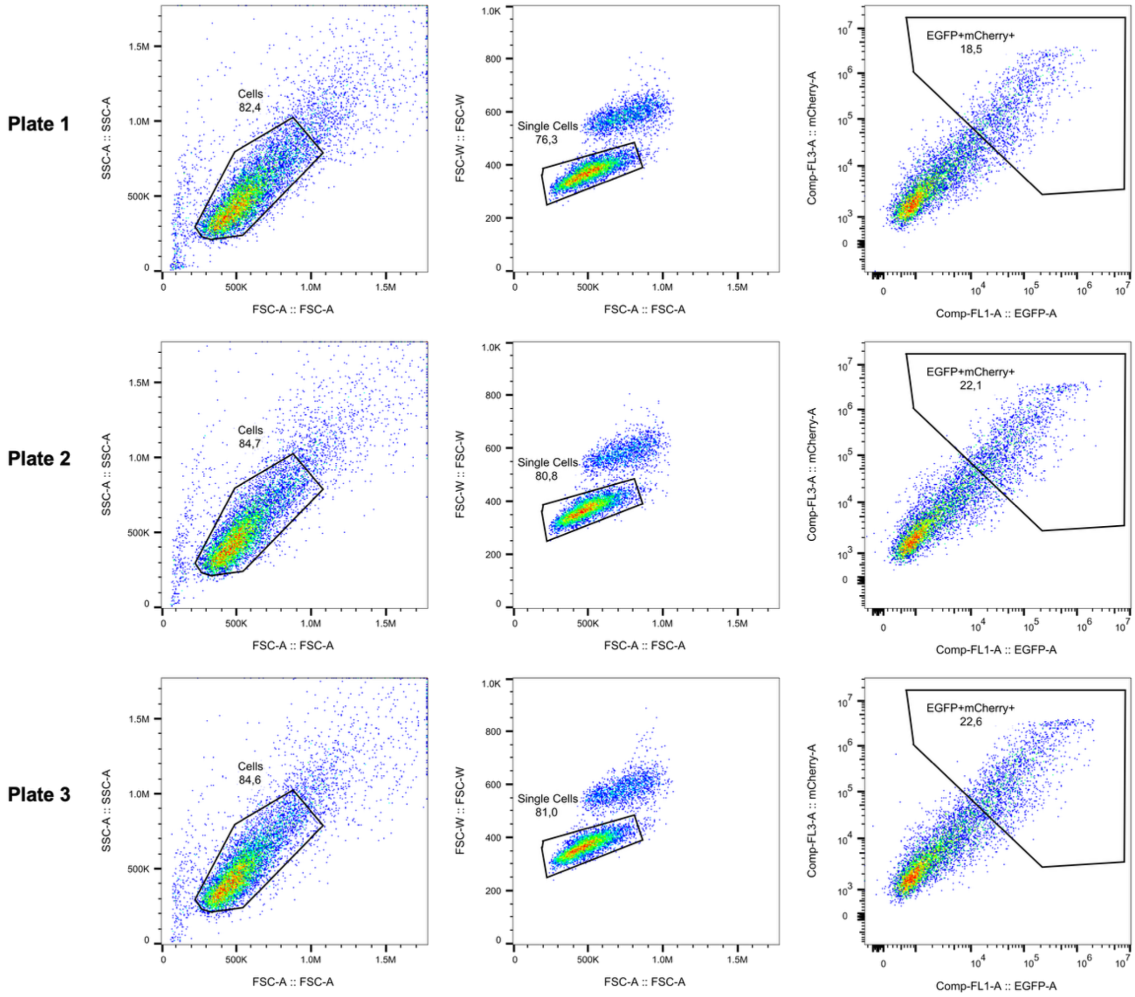

		All events	Cells	Single cells*	EGFP+mCherry+*
WT + NAT16-V5	Plate 1	100 %	82.4 %	76.3 %	18.5 %
	Plate 2	100 %	84.7 %	80.8 %	22.1 %
	Plate 3	100 %	84.6 %	81.0 %	22.6 %
	**Mean**	**100 %**	**83.9 %**	**79.4 %**	**21.1 %**
	**Standard deviation**	**0 %**	**1.30 %**	**2.66 %**	**2.24 %**

b) *NAT16* KO + NAT16-V5

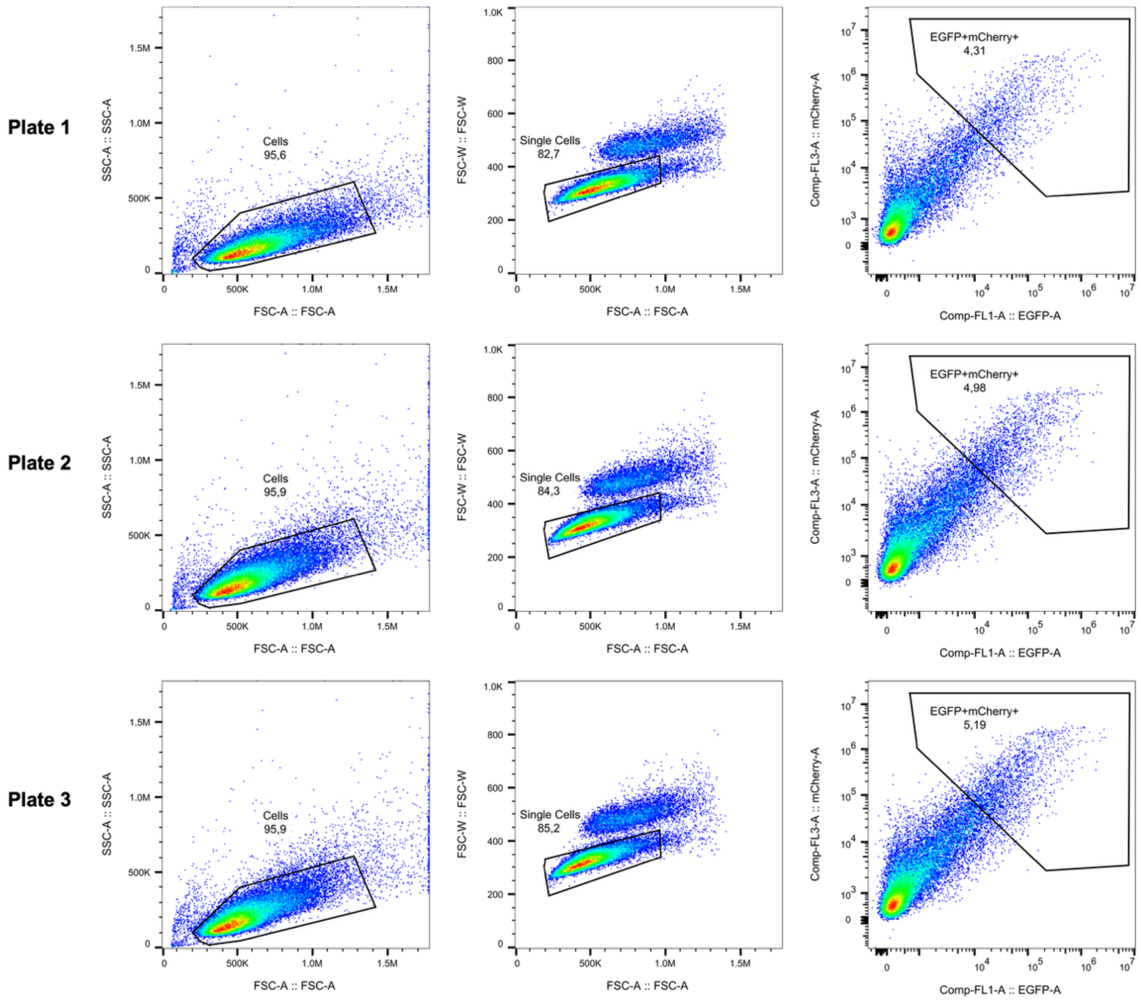

		All events	Cells	Single cells*	EGFP+mCherry+*
NAT16 KO + NAT16-V5	Plate 1	100 %	95.6 %	82.7 %	4.31 %
	Plate 2	100 %	95.9 %	84.3 %	4.98 %
	Plate 3	100 %	95.9 %	85.2 %	5.19 %
	**Mean**	**100 %**	**95.8 %**	**84.1 %**	**4.83 %**
	**Standard deviation**	**0 %**	**0.17 %**	**1.27 %**	**0.46 %**

Supplementary Figure 13. Fluorescence-activated cell sorting (FACS). FACS of NAT16 overexpression cell lines with a) WT and b) *NAT16* KO background. First, the main population of cells were gated based on cell size and granularity using Forward Scatter Area (FSC-A) and Side Scatter Area (SSC-A). Single cells were gated for using FSC-A and Forward Scatter Width (FSC-W), and from the single cells, we gated for the combination of both EGFP- and mCherry-signal. Numbers for each gate represent percentages of the frequency of parent gate, also shown in the representative tables below the plots.

Supplementary Table 1. Results from DTNB screening with Ac-CoA as co-substrate. All amino acids were the L- stereoisomer unless indicated otherwise. Source data are provided as a Source Data file.

Peptides	CoA (µM)	St. dev.
MLGPEGGRWGRPVGRRRRPVRVYP	-0.5	0.8
MDELFPLRWGRPVGRRRRPVRVYP	-0.3	1.0
EEEIAALRWGRPVGRRRRPVRVYP	-2.0	2.3
SESSSKSRWGRPVGRRRRPVRVYP	0.3	1.0
DDDIAALRWGRPVGRRRRPVRVYP	-1.6	2.7
MTNKSSLKNN	-2.1	1.9
MAPLDLDRWGRPVGRRRRPVRVYP	-3.7	0.9
GGQISKGFGKLY	1.0	1.5
GHALCVCSRGTV	-13.9	10.2
GHALSVSSRGTV	1.7	0.5
HEALSEARWGRPVGRRRRPVRVYP	1.4	1.0
SEALSEARWGRPVGRRRRPVRVYP	-0.5	0.5
HLSVNDLRWGRPVGRRRRPVRVYP	-1.5	1.1
SLSVNDLRWGRPVGRRRRPVRVYP	-1.4	0.6
HSLLPVDRWGRPVGRRRRPVRVYP	-0.4	0.4
SSLLPVDRWGRPVGRRRRPVRVYP	-0.6	0.6

Amino acids	CoA (µM)	St. dev.
Alanine	1.8	3.4
Arginine	189.7	5.2
Asparagine	-0.5	0.4
Aspartate	2.1	1.8
Citrulline	3.3	1.6
Cysteine	7.8	22.8
Cystine	-2.2	0.5
Glutamate	1.2	2.1
Glutamine	0.3	2.4
Glycine	0.3	3.8
Histidine	>300	4.7
Hydroxyproline	-3.0	1.7
Isoleucine	-1.0	4.1
Leucine	-4.5	2.1
Lysine	73.5	4.2
Methionine	13.2	2.0
Ornithine	43.2	3.0
Phenylalanine	70.0	6.4
Proline	3.9	3.2
Serine	-2.0	0.7
Threonine	0.9	0.6
Tryptophan	-0.9	0.3
Tyrosine	16.1	2.0
Valine	4.7	3.1

Histidine derivatives	CoA (µM)	St. dev.
1-Methyl-L-Histidine	>300	2.3
3-Methyl-L-Histidine	>300	1.0
Urocanic acid	1.3	1.4
D-Histidine	1.2	0.3
Histamine	-1.0	1.3
Imidazole propionic acid	0.2	0.9
L-Carnosine	0.9	0.6
L-Histidinol	8.1	0.9

Other	CoA (µM)	St. dev.
4-Aminobenzoic acid	-3.1	0.3
Agmatine	1.6	0.6
Allantoin	0.7	0.7
Creatine	1.9	2.3
Cytidine	4.0	9.8
D-glucosamine	-4.8	1.0
Dopamine	0.1	3.5
Guanosine	-3.5	0.9
L-homoserine lactone	-1.0	1.6
Nicotinamide	-0.7	2.0
Serotonin	1.7	1.3
Tyramine	3.7	0.8
Urea	0.3	1.9

Supplementary Table 2. Crystal data collection and data processing statistics for unmerged anomalous data of the iodide derivative datasets. Values in parenthesis are for the highest resolution shell.

Crystal	NAT16_49	NAT16_52-1	NAT16_52-2	Combined dataset
Beamline	P11, PETRA III	P11, PETRA III	P11, PETRA III	
Detector	Pilatus 6M-F	Pilatus 6M-F	Pilatus 6M-F	
Temperature (K)	100	100	100	
Wavelength (Å)	2.07	2.07	2.07	
Resolution range (Å)	50-2.6 (2.75-2.6)	50-2.6 (2.75-2.6)	50-2.6 (2.75-2.6)	50-2.6 (2.66-2.6)
Space group	$P\ 6_3$	$P\ 6_3$	$P\ 6_3$	$P\ 6_3$
Unit cell (Å)<br>(°)	90.8 90.8 99.2<br>90 90 120	90.6 90.6 99.1<br>90 90 120	90.6 90.6 99.1<br>90 90 120	90.6 90.6 99.1<br>90 90 120
Total reflections	300599 (33198)	300765 (33066)	300446 (33286)	901247 (37289)
Unique reflections	28171 (4467)	27967 (4398)	27975 (4410)	28049 (2056)
Multiplicity	10.7 (7.4)	10.8 (7.5)	10.7 (7.5)	32.1 (18.1)
Completeness (%)	99.6 (97.7)	99.3 (96.1)	99.4 (96.3)	99.7 (97.4)
Mean *I*/sigma(*I*)	7.58 (0.61)	5.73 (0.59)	5.51 (0.47)	10.62 (0.77)
R_{meas}	0.28 (3.13)	0.46 (2.71)	0.53 (3.11)	0.45 (3.41)
$CC_{1/2}$ (%)	99.4 (22.6)	98.8 (41.6)	98.6 (31.1)	95.0 (47.7)

Supplementary Table 3. HisAT crystallization and cryo-protection conditions.

Crystal	Protein buffer	Crystallization well solution	Cryo solution
NAT16_49	10 mM Tris-HCl pH 7.5, 150 mM NaCl, 10% glycerol, 1 mM L-histidine, 0.5 mM CoA	0.1 M MIB buffer pH 8.0, 25% PEG 1500	20% glycerol, 76% well solution and 0.2 M NaI
NAT16_52	10 mM Tris-HCl pH 7.5, 150 mM NaCl, 10% glycerol, 1 mM L-histidine, 0.5 mM CoA	0.1 M MIB buffer pH 8.0, 25% PEG 1500	20% glycerol, 76% well solution and 0.2 M NaI
NAT16 d5-27_2 (9emd)	10 mM Tris-HCl pH 7.5, 150 mM NaCl, 10% glycerol, 1 mM L-histidine, 0.7 mM CoA	0.1 M MIB buffer pH 7.0, 25 % w/v PEG 1500	60% well solution, 20% Glycerol, 20% PEG 1500
NAT16 d5-45_9 (9emo)	10 mM Tris-HCl pH 7.5, 150 mM NaCl, 10% glycerol, 1 mM L-arginine, 0.7 mM Ac-CoA	0.1 M Bis-tris pH 6.8, 20% PEG 3350	0.1 M Bis-tris pH 6.8, 20% PEG 3350, 20% Glycerol, 1 mM L-arginine, 1 mM Ac-CoA
NAT16 d5-45_18 (9emp)	10 mM Tris-HCl pH 7.5, 150 mM NaCl, 10% glycerol, 1 mM L-histidine, 0.7 mM Myr-CoA	0.1 M Bis-tris pH 6.8, 22% PEG3350	0.1 M Bis-tris pH 6.8, 22% PEG3350, 20% Glycerol
NAT16 d5-27_42 (9emt)	10 mM Tris-HCl pH 7.5, 150 mM NaCl, 10% glycerol, 2 mM adenosine, 1 mM Ac-CoA	0.1 M MIB pH 6.5, 20% PEG3350	75% well solution, 25% glycerol
NAT16 d5-45_49 (9en3)	10 mM Tris-HCl pH 7.5, 150 mM NaCl, 10% glycerol, 0.7 mM S-Ethyl-CoA, 0.7 mM L-histidine	0.1 M HEPES pH 7, 18% PEG 3000	0.1 M Bis-tris pH 6.8, 20% PEG 3000, 25% glycerol, 1 mM S-Ethyl-CoA, 0.7 mM L-histidine

MIB = malonate: imidazole: boric acid buffer (2:3:3).

Supplementary Table 4. HisAT activity was studied in the presence of histidine and different Acyl-CoA molecules using a DTNB assay. Acyl-CoA concentration was 300 µM, indicating that both propionyl-CoA and butyryl-CoA (like Ac-CoA) were completely transformed into CoA during the 30-minute reaction. Source data are provided as a Source Data file.

Acyl-CoA	CoA (µM)	St. dev.
Propionyl-CoA	>300	31.5
Butyryl-CoA	>300	3.6
Palmitoyl-CoA	143.7	3.9
Oleoyl-CoA	71.5	2.0
Arachidonoyl-CoA	60.0	1.4
Malonyl-CoA	12.4	1.1
Succinyl-CoA	11.0	8.2
Glutaryl-CoA	35.7	1.9

Supplementary Table 5. Results from DTNB screening with Myristoyl-CoA as co-substrate. All amino acids were the L- stereoisomer unless indicated otherwise. Source data are provided as a Source Data file.

Peptides	CoA (µM)	St. dev.
GGQISKGFGKLY	0.1	0.9
GHALCVCSRGTV	15.7	7.0
GHALSVSSRGTV	-0.7	1.1
VPKPEKKTARDA	1.0	1.1
RKEKDKKGCHCV	-1.3	2.8
EEALPKKTGGPQ	-0.6	1.9
NGKVLKKRRLSL	0.7	1.7
VPAGATSVDRLV	-1.4	0.5

Amino acids	CoA (µM)	St. dev.
Alanine	0.3	1.0
Arginine	1.9	0.3
Glycine	-1.5	0.4
Histidine	241.1	9.0
Lysine	-0.4	0.7
Phenylalanine	0.9	0.7
Tryptophan	-0.6	1.0
Tyrosine	-0.5	1.3

Other	CoA (µM)	St. dev.
Dopamine	-3.6	1.3
Ethanolamine	-0.4	1.7

Supplementary Table 6. Melting temperatures from the differential scanning fluorimetry (DSF) analysis of HisAT in the presence of CoA or CoA derivatives. The highlighted conditions with the higher concentrations of myristoyl-, palmitoyl-, and oleoyl-CoA were unreliable, probably because of micelle formation. The mean of the melting temperature (T_m) from three parallel measurements and the standard deviation are reported. Source data are provided as a Source Data file.

Additive	Concentration	T_m	St.Dev.
water		38.2	0.06
CoA	10 µM	38.2	0.06
CoA	100 µM	38.9	0.12
Ac-CoA	10 µM	38.4	0.15
Ac-CoA	100 µM	40.0	0.17
Propionyl-CoA	10 µM	38.7	0.06
Propionyl-CoA	100 µM	40.8	0.4
Butyryl-CoA	10 µM	38.6	0.1
Butyryl-CoA	100 µM	41.1	0.06
Myristoyl-CoA	10 µM	41.6	1.4
Myristoyl-CoA	100 µM	33.4	16.71
Palmitoyl-CoA	10 µM	39.8	0.75
Palmitoyl-CoA	100 µM	35.5	9.54
Oleoyl-CoA	10 µM	39.0	0.1
Oleoyl-CoA	100 µM	33.3	11.49
Arachidonoyl-CoA	10 µM	39.5	0.2
Arachidonoyl-CoA	100 µM	40.6	0.23
Glutaryl-CoA	10 µM	37.9	0.06
Glutaryl-CoA	100 µM	38.2	0.15
Malonyl-CoA	10 µM	37.9	0.06
Malonyl-CoA	100 µM	38.4	0.1
Succinyl-CoA	10 µM	38.0	0.1
Succinyl-CoA	100 µM	38.6	0.06

Supplementary Table 7. NAT16 and neighboring sequences for species in different mammalian orders. The phylogenetic tree is based on https://pubmed.ncbi.nlm.nih.gov/11743200/. Total number of species with NAT16-like sequences and sequences presented as 'Low quality protein' or significantly truncated (LQP) from the Refseq database.

	NAT16		MOGAT3		VGF	
Mammalian order	Total	LQP	Total	LQP	Total	LQP
Cetartiodactyla	41	41	34	19	42	4
Perissodactyla	6	6	5	5	5	1
Carnivora	40	39	43	0	45	1
Pholidota	2	2	1	1	2	0
Chiroptera	23	20	16	1	24	0
Euliotyphla	6	6	6	0	6	1
Rodentia	10	10	16	4	42	2
Lagomorpha	1	1	4	1	4	0
Dermoptera	2	2	2	0	2	0
Scandentia	1	0	1	0	1	0
Primates	33	4	34	0	32	4
Pilosa	1	0	1	0	1	0
Cingulata	1	0	0	0	1	0
Afrosoricida	2	0	1	0	2	0
Macroscelidea	1	0	1	0	1	0
Tubulidentata	1	0	1	0	1	0
Sirenia	1	1	0	0	1	0
Hyracoidea	0	0	0	0	0	0
Proboscidea	2	2	0	0	2	0
Marsupialia	0	0	7	1	8	1
Monotremata	2	2	2	0	1	1
Overall	176	136	175	32	223	15

Supplementary Table 8. List of proteins included in the structural similarity analysis.

#	Uniprot ID	PDB ID	Species	Protein Name	Gene Name
1	Q8N8M0	9en3	*Homo sapiens*	Histidine acetyltransferase	*NAT16*
2	P30419	5o9u	*Homo sapiens*	Glycylpeptide N-tetradecanoyltransferase 1	*NMT1*
3	O60551		*Homo sapiens*	Glycylpeptide N-tetradecanoyltransferase 2	*NMT2*
4	E7FET0		*Danio rerio*	N-acetyltransferase 16	*nat16*
5	F1QKD7		*Danio rerio*	N-acetyltransferase 16,-like	*nat16l*
6	B8JIQ9		*Danio rerio*	Glycylpeptide N-tetradecanoyltransferase	*nmt1b*
7	A7YT82		*Danio rerio*	Glycylpeptide N-tetradecanoyltransferase 2	*nmt2*
8	O61613		*Drosophila melanogaster*	Glycylpeptide N-tetradecanoyltransferase	*Nmt*
9	Q9FN10		*Arabidopsis thaliana*	Acyl-CoA N-acyltransferases (NAT) superfamily protein	
10	O64737		*Arabidopsis thaliana*	Acyl-CoA N-acyltransferases (NAT) superfamily protein	
11	O64815		*Arabidopsis thaliana*	Probable N-acetyltransferase HLS1-like	
12	Q42381		*Arabidopsis thaliana*	Probable N-acetyltransferase HLS1	*HLS1*
13	Q9LTR9		*Arabidopsis thaliana*	Glycylpeptide N-tetradecanoyltransferase 1	*NMT1*
14	Q94L32		*Arabidopsis thaliana*	Putative glycylpeptide N-tetradecanoyltransferase 2	*NMT2*
15	Q54G61		*Dictyostelium discoideum*	GCN5-related N-acetyltransferase	
16	Q553B6		*Dictyostelium discoideum*	Glycylpeptide N-tetradecanoyltransferase	*nmt*
17	Q54LZ9		*Dictyostelium discoideum*	N-acetyltransferase domain-containing protein	
18	Q54ML0		*Dictyostelium discoideum*	N-acetyltransferase domain-containing protein	
19	P14743	2nmt	*Saccharomyces cerevisiae*	Glycylpeptide N-tetradecanoyltransferase	*NMT1*
20	Q8ILW6		*Plasmodium falciparum*	Glycylpeptide N-tetradecanoyltransferase	*NMT*
21	A0A5A8CVU3		*Cafeteria roenbergensis* (Marine flagellate)	N-acetyltransferase domain-containing protein	
22	A0A5A8DTB6		*Cafeteria roenbergensis* (Marine flagellate)	Glycylpeptide N-tetradecanoyltransferase	
23	F2UNI0		*Salpingoeca rosetta* (strain ATCC 50818 / BSB-021)	N-acetyltransferase domain-containing protein	
24	E1Z5X7		*Chlorella variabilis* (Green alga)	N-acetyltransferase domain-containing protein	*CHLNCDRAFT_ 140669*
25	A0A2V3IER9		*Gracilariopsis chorda*	Uncharacterized protein	*BWQ96_09722*
26	R7QTB4		*Chondrus crispus* (Carrageen Irish moss)	N-acetyltransferase domain-containing protein	*CHC_ T00000861001*
27	A0A7S4HZQ5		*Vannella sp.* CB-2014 / Vannella robusta	Hypothetical protein	*VSP0166_ LOCUS6528*
28	Q9HJZ0	3c26	*Thermoplasma acidophilum*	N-acetyltransferase domain containing protein	
29	Q8U286		*Pyrococcus furiosus* (strain ATCC 43587 / DSM 3638 / JCM 8422 / Vc1)	N-acetyltransferase domain-containing protein	
30	A0A524CVK5		*Thorarchaeota archaeon* (strain OWC)	GNAT family N-acetyltransferase	
31	A0A7K4IQS1		*Candidatus Bathyarchaeota archaeon*	GNAT family N-acetyltransferase	*HXY33_07145*
32	M0DDK8		*Halogeometricum pallidum* JCM 14848	N-acetyltransferase domain-containing protein	*C474_06150*
33	L9XG87		*Natronococcus amylolyticus* DSM 10524	N-acetyltransferase GCN5	*C491_04976*
34	A0A097QWM5		*Thermococcus eurythermalis*	GNAT family acetyltransferase	*TEU_11460*
35	O57768	3ddd	*Pyrococcus horikoshii* (strain ATCC 700860 / DSM 12428 / JCM 9974 / NBRC 100139 / OT-3)	N-acetyltransferase domain-containing protein	
36	A0A2E6C328		*Euryarchaeota archaeon*	Glycylpeptide N-tetradecanoyltransferase	*CMB57_06290*
37	A0A3L6J856		*Thorarchaeota archaeon* (strain OWC)	N-acetyltransferase domain-containing protein	*C4K49_12200*
38	A0A6G2HH32		*Halorubrum sp.* JWXQ-INN 858	GNAT family N-acetyltransferase	*GRS48_11315*
39	Q8KRB5	2wpx	*Streptomyces clavuligerus*	clavulanic acid pathway acetyltransferase	*SCLAV_4185*
40	Q5C8M4	7wx7	*Legionella pneumophila*	GNAT family N-acetyltransferase	*vipF*
41	P9WJM7	1ozp	*Mycobacterium tuberculosis*	Mycothiol acetyltransferase	*mshD*
42	A0A2W6C360		*Chloroflexi bacterium*	N-acetyltransferase domain-containing protein	*DLM70_06705*
43	A0A398D2B0		*Candidatus Cryosericum odellii*	GNAT family N-acetyltransferase	
44	A0A523V9J1		*Anaerolineales bacterium*	GNAT family N-acetyltransferase	*E3J64_06845*
45	A0A5D0SFC2		*Kosmotoga sp*	GNAT family N-acetyltransferase	*FXF54_08220*
46	A0A7Y2F0T9		*Acidimicrobiia bacterium*	GNAT family N-acetyltransferase	*HKN46_08360*
47	F0SSH4		*Rubinisphaera brasiliensis* (strain ATCC 49424 / DSM 5305 / JCM 21570 / NBRC 103401 / IFAM 1448) (*Planctomyces brasiliensis*)	Uncharacterized protein	*Plabr_1634*
48	A0A1F9AMY8		*Deltaproteobacteria bacterium* RBG_13_61_14	N-acetyltransferase domain-containing protein	*A2V67_19930*
49	A0A536FZ46		*Chloroflexi bacterium / Chloroflexota bacterium*	GNAT family N-acetyltransferase	*E6I26_14100*
50	Q969I3		*Homo sapiens*	Glycine N-acyltransferase-like protein 1	*GLYATL1*
51	Q8WU03		*Homo sapiens*	Glycine N-acyltransferase-like protein 2	*GLYATL2*
52	Q5SZD4		*Homo sapiens*	Glycine N-acyltransferase-like protein 3	*GLYATL3*
53	Q2KIR7	7pk0	*Bos taurus*	Glycine N-acyltransferase	*GLYAT*
54	P9WFK7	3r1k	*Mycobacterium tuberculosis* (strain ATCC 25618 / H37Rv)	N-acetyltransferase Eis	*eis*
55	A0QY29	3sxn	*Mycolicibacterium smegmatis* (strain ATCC 700084 / mc(2)155) (*Mycobacterium smegmatis*)	Enhanced intracellular survival protein	

Supplementary Table 9. Polar and semi-polar method details for metabolomics LC/MS analysis.

	Polar method details
LC conditions	
LC system:	Thermo Scientific Vanquish
Column:	InfinityLab Poroshell 120 HILIC-Z 2.1x150 mm, 2.7 µm
Flow rate:	250 µl/min
Column temperature:	30°C
Mobile Phase A:	10 mM ammonium acetate in 90% Acetonitrile
Mobile Phase B:	10 mM ammonium acetate in ultrapure water
Gradient:	0.0 min 10% B
	2.0 min 10% B
	14.0 min 40% B
	15.0 min 40% B
	16.0 min 10% B
	24.0 min 10% B
Injection volume:	5 µl
MS conditions	
MS system:	Thermo Q Exactive HF MS
Ionization mode:	ESI +/-
Capillary temperature:	250°C
Acquisition mode:	Full Scan
Scan range:	65 – 975 m/z
Resolution:	120.000
	Semi-polar method details
LC conditions	
LC system:	Thermo Scientific Vanquish
Column:	ACQUITY HSS T3 2.1x150 mm, 1.8 µm
Flow rate:	300 µl/min
Column temperature:	30°C
Mobile Phase A:	10 mM ammonium formate, 0.1% formic acid in water
Mobile Phase B:	10 mM ammonium formate, 0.1% formic acid in methanol
Gradient:	0.0 min 0% B
	2.0 min 0% B
	4.0 min 35% B
	6.0 min 90% B
	14.0 min 90% B
	14.1 min 0% B
	15.0 min 0% B
Injection volume:	5 µl
MS conditions	
MS system:	Thermo Exploris 240
Ionisation mode:	Polarity switching
Capillary temperature:	320°C
Acquisition mode:	Full Scan
Scan range:	65 – 975 m/z
Resolution:	120.000
