# Supplementary material for: The molecular basis for acetylhistidine synthesis by HisAT/NAT16
Source: Nat Commun. 2025 Jul 1;16:5960. doi: 10.1038/s41467-025-61145-x (PMC12219266; doi:10.1038/s41467-025-61145-x)
Supplement: Supplementary file 2 — Description of Additional Supplementary Files [file 41467_2025_61145_MOESM2_ESM.pdf]

Description of Additional Supplementary Files

File name: Supplementary Data 1
Description: Results from the HAP1 cell metabolomics analysis. DP = Descriptive power, LOD = limit of detection, RT = retention time. Annotations on level 1 are based on accurate mass, MSMS spectra and known retention time obtained from standards analyzed on the same system, annotations on level 2a are based on accurate mass and known retention time as obtained from standards analyzed on the same system, and annotations on level 2b are based on accurate mass and MSMS spectra from an external library. Normality data distribution was checked by Shapiro-Wilk test to select the best-suited univariate statistical test. T-tests were performed because the data follow a normal distribution. The Benjamini-Hochberg critical value at false positive rate 0.05 was used to adjust the p-values to correct for the high number of variables. The adjusted p-values with pink background were below 0.05.
