# Supplementary material for: The molecular basis for acetylhistidine synthesis by HisAT/NAT16
Source: Nat Commun. 2025 Jul 1;16:5960. doi: 10.1038/s41467-025-61145-x (PMC12219266; doi:10.1038/s41467-025-61145-x)
Supplement: Supplementary file 4 — Reporting Summary [file 41467_2025_61145_MOESM4_ESM.pdf]

nature portfolio

Corresponding author(s): Matti Myllykoski

Last updated by author(s): Jun 13, 2025

Reporting Summary

Nature Portfolio wishes to improve the reproducibility of the work that we publish. This form provides structure for consistency and transparency in reporting. For further information on Nature Portfolio policies, see our Editorial Policies and the Editorial Policy Checklist.

Statistics

For all statistical analyses, confirm that the following items are present in the figure legend, table legend, main text, or Methods section.

n/a	Confirmed	
☐	☒	The exact sample size (*n*) for each experimental group/condition, given as a discrete number and unit of measurement
☐	☒	A statement on whether measurements were taken from distinct samples or whether the same sample was measured repeatedly
☐	☒	The statistical test(s) used AND whether they are one- or two-sided<br>*Only common tests should be described solely by name; describe more complex techniques in the Methods section.*
☒	☐	A description of all covariates tested
☐	☒	A description of any assumptions or corrections, such as tests of normality and adjustment for multiple comparisons
☐	☒	A full description of the statistical parameters including central tendency (e.g. means) or other basic estimates (e.g. regression coefficient) AND variation (e.g. standard deviation) or associated estimates of uncertainty (e.g. confidence intervals)
☐	☒	For null hypothesis testing, the test statistic (e.g. *F*, *t*, *r*) with confidence intervals, effect sizes, degrees of freedom and *P* value noted<br>*Give P values as exact values whenever suitable.*
☒	☐	For Bayesian analysis, information on the choice of priors and Markov chain Monte Carlo settings
☒	☐	For hierarchical and complex designs, identification of the appropriate level for tests and full reporting of outcomes
☒	☐	Estimates of effect sizes (e.g. Cohen's *d*, Pearson's *r*), indicating how they were calculated

Our web collection on statistics for biologists contains articles on many of the points above.

Software and code

Policy information about availability of computer code

Data collection	i-control v. 3.9.1.0 (Tecan Infinite M Nano plate reader),<br>Sparkcontrol magellan v. 2.2 (Tecan SPARK 20M),<br>Image Lab 6.0.1 (Chemidoc XRS+, western blot images,<br>Image Lab 6.0.0 (Gel Doc EZ, gel images),<br>Unicorn 7.1 (Äkta pure),<br>Compound Discoverer 3.3 (Thermo Scientific), and<br>Skyline 22.2 (MacCross Lab Software)(metabolomics),<br>Human Protein Atlas v.23 (tissue & cell expression levels),<br>CrystalControl (crystal data collection, Beamline P11),<br>mxCuBE v2 (crystal data collection, Beamline P14),<br>Dali server (http://ekhidna2.biocenter.helsinki.fi/dali/) and<br>Foldseek (https://search.foldseek.com/search) (structure similarity searches),<br>Bio-Rad CFX Maestro 1.1 version 4.1.2433.1219 (DSF),<br>FlowJo v. 10 (Flow cytometry),<br>DISOPRED3 as part of PSIPRED (http://bioinf.cs.ucl.ac.uk/psipred/) (disorder prediction),<br>DELTA-BLAST (sequence similarity search).
Data analysis	XDS v. Jan 31, 2020, Feb 5, 2021, and Jan 10, 2022 (crystal data processing),<br>SHELXC v. 2016/1, SHELXD v. 2013/2, SHELXE v. 2019/1 (structure solution),

PHENIX v. 1.20.1_4487 (structure refinement & analysis), PHENIX autobuild (structure solution), PHENIX.phaser v. 2.8.3 (molecular replacement),
Wincoot v. 0.9.8.7 (structure refinement),
PyMOL v. 2.5.5 and 3.0.4 (structure visualization),
Dali (http://ekhidna2.biocenter.helsinki.fi/dali/) (structure similarity analysis),
Archaeopteryx v. 0.9930 (dendrogram visualization),
MAFFT v. 7 (https://mafft.cbrc.jp/alignment/software/) (sequence alignment),
Unipro UGENE v. 50.0 (sequence alignment visualization),
ICEKAT server (https://icekat.herokuapp.com/icekat) (enzyme kinetics analysis),
DSFWorld (https://gestwickilab.shinyapps.io/dsfworld/) (DSF data analysis),
MatLab (2022b, MathWorks) (metabolomics annotation),
Microsoft 365 Excel (simple calculations and data handling),
Graphpad Prism 10 (graph generation, enzyme kinetics analysis),
Inkscape v. 1.2 (figure assembly),
Adobe Illustrator v. 29.4 (figure assembly).

For manuscripts utilizing custom algorithms or software that are central to the research but not yet described in published literature, software must be made available to editors and reviewers. We strongly encourage code deposition in a community repository (e.g. GitHub). See the Nature Portfolio guidelines for submitting code & software for further information.

Data

Policy information about availability of data

All manuscripts must include a data availability statement. This statement should provide the following information, where applicable:

- Accession codes, unique identifiers, or web links for publicly available datasets
- A description of any restrictions on data availability
- For clinical datasets or third party data, please ensure that the statement adheres to our policy

The crystal structures reported in this manuscript have been deposited to Protein Data Bank with identifiers 9emd, 9emo, 9emp, 9emt, and 9en3 (see Table 1). The enzyme kinetics assay protocols and results have been deposited to STRENDA database with DOI 10.22011/strenda_db.HYQ0CA and 10.22011/strenda_db.ZT1UDP. The metabolomics data has been deposited to MetaboLights database with the identifier MTBLS12070. Source data for the figures with graphs and tables is included in the source data file.

Research involving human participants, their data, or biological material

Policy information about studies with human participants or human data. See also policy information about sex, gender (identity/presentation), and sexual orientation and race, ethnicity and racism.

Reporting on sex and gender	n/a
Reporting on race, ethnicity, or other socially relevant groupings	n/a
Population characteristics	n/a
Recruitment	n/a
Ethics oversight	n/a

Note that full information on the approval of the study protocol must also be provided in the manuscript.

Field-specific reporting

Please select the one below that is the best fit for your research. If you are not sure, read the appropriate sections before making your selection.

☒ Life sciences ☐ Behavioural & social sciences ☐ Ecological, evolutionary & environmental sciences

For a reference copy of the document with all sections, see nature.com/documents/nr-reporting-summary-flat.pdf

Life sciences study design

All studies must disclose on these points even when the disclosure is negative.

Sample size	No statistical methods were used to pre-determine sample size, but our sample size is according to conventions in the field. For small-scale experiments, the number of replicates exceeds at least 3 biologically independent experiments and/or at least 3 technical replicates, as indicated in each figure legend and/or in the Methods.
Data exclusions	When the secondary plots were used to determine the KM and VMax values, only the apparent values determined at lower Ac-CoA concentrations were used because higher Ac-CoA concentrations inhibited the reaction resulting in non-linear curves. All technically sound data were included.

Replication	Each substrate in the substrate screening assay was measured once. Cell fractionation assays were repeated three times independently, and the enzyme kinetics assays were repeated at least three times. Metabolomics assays were based on five replicates (separate dishes of cells) for each of the four sample groups. Differential scanning fluorimetry assays had three technical replicates for each condition. We present no experimental results that were not reproducible.
Randomization	Covariates were not relevant. No randomization was performed in this study, due to small sample size.
Blinding	For metabolomics analysis, blinding was not done since the small sample groups have to be defined during data analysis. Determination of significance was solely based on automated procedures. For immunoblotting and enzyme assays blinding was not done since samples were prepared and analyzed by the same researcher in small batches.

Reporting for specific materials, systems and methods

We require information from authors about some types of materials, experimental systems and methods used in many studies. Here, indicate whether each material, system or method listed is relevant to your study. If you are not sure if a list item applies to your research, read the appropriate section before selecting a response.

Materials & experimental systems

n/a	Involved in the study
☐	☒ Antibodies
☐	☒ Eukaryotic cell lines
☒	☐ Palaeontology and archaeology
☒	☐ Animals and other organisms
☒	☐ Clinical data
☒	☐ Dual use research of concern
☒	☐ Plants

Methods

n/a	Involved in the study
☒	☐ ChIP-seq
☐	☒ Flow cytometry
☒	☐ MRI-based neuroimaging

Antibodies

Antibodies used	Anti-V5 (Invitrogen, 46-1157 (same as R960CUS), clone SV5-Pk1, lot 3045095, dilution 1:1000)<br>Anti-Histone H4 (Millipore, 05-858, clone 62-141-13, lot not known, dilution 1:3000)<br>Anti-Calnexin (Abcam, ab133615, clone EPR3633(2), lot 1036718-18, dilution 1:500)<br>Anti-GAPDH (Santa Cruz, Sc-47724, clone 0411, lot G2920, dilution 1:1000)<br><br>Anti-Rabbit (Cytiva, GENA934-1ML, lot 14187089, dilution 1:3000)<br>Anti-Mouse (Cytiva, GENA931-1ML, lot 17162269, dilution 1:3000)
Validation	Primary antibodies were listed in the Cell fractionation section of the Methods chapter. These were validated based on their molecular weights and are commercially available and tested by the manufacturers.

Eukaryotic cell lines

Policy information about cell lines and Sex and Gender in Research

Cell line source(s)	HAP1 WT cells (clone C631; sex: male; RRID: CVCL Y019) and the NAT16 KO cells (HZGHC007792c003) were obtained from Horizon Discovery. Sf9 cells were a gift from another research group in the facility and were originally obtained from Thermo Scientific (11496015).
Authentication	For HAP1 WT and NAT16 KO cells, total RNA was isolated, reverse transcribed and amplified with NAT16-specific primers. Sf9 cells were not authenticated.
Mycoplasma contamination	HAP1 cell lines were tested negative for mycoplasma contamination by DAPI staining. Sf9 cells were not tested for mycoplasma contamination.
Commonly misidentified lines<br>(See ICLAC register)	No commonly misidentified cell lines were used in the study.

Plants

Seed stocks

Report on the source of all seed stocks or other plant material used. If applicable, state the seed stock centre and catalogue number. If plant specimens were collected from the field, describe the collection location, date and sampling procedures.

Novel plant genotypes

Describe the methods by which all novel plant genotypes were produced. This includes those generated by transgenic approaches, gene editing, chemical/radiation-based mutagenesis and hybridization. For transgenic lines, describe the transformation method, the number of independent lines analyzed and the generation upon which experiments were performed. For gene-edited lines, describe the editor used, the endogenous sequence targeted for editing, the targeting guide RNA sequence (if applicable) and how the editor was applied.

Authentication

Describe any authentication procedures for each seed stock used or novel genotype generated. Describe any experiments used to assess the effect of a mutation and, where applicable, how potential secondary effects (e.g. second site T-DNA insertions, mosiacism, off-target gene editing) were examined.

Flow Cytometry

Plots

Confirm that:

☒ The axis labels state the marker and fluorochrome used (e.g. CD4-FITC).

☒ The axis scales are clearly visible. Include numbers along axes only for bottom left plot of group (a 'group' is an analysis of identical markers).

☒ All plots are contour plots with outliers or pseudocolor plots.

☒ A numerical value for number of cells or percentage (with statistics) is provided.

Methodology

Sample preparation

Forty-eight hours post transfection, the cells were prepped for single cell sorting into three 96 well plates (Sarstedt #83.3924.500) containing 200 µl 20 % FBS, 1 % Penicillin-Streptomycin, IMDM cell medium. They were trypsinated (Gibco, # 25300054), washed in 1x Phosphate buffered saline solution (PBS, Gibco, #18912014, 1xPBS), harvested at 300 x g for 5 min, and finally resuspended in roughly 3 ml IMDM.

Instrument

Sony SH800 cell sorter

Software

FlowJo v. 10

Cell population abundance

Describe the abundance of the relevant cell populations within post-sort fractions, providing details on the purity of the samples and how it was determined.

Gating strategy

Background signal was detected in non-transfected cells and cells transfected with either of the two plasmids to determine the gating of target cells. Cell sorting was done with the lowest flow pressure possible, aiming for a maximum of 500 cells per second. First, the main population of cells were gated based on cell size and granularity using Forward Scatter Area (FCS-A) and Back Scatter Area (BSC-A). Single cells were gated for using FSC-A and Forward Scatter Width (FSC-W), and from the single cells, we gated for the combination of both EGFP- and mCherry-signal. EGFP- and mCherry-containing cells were detected with filter FL1 (525/50) and FL3 (617/30), respectively.

☒ Tick this box to confirm that a figure exemplifying the gating strategy is provided in the Supplementary Information.
